# Essential role of IκB_NS_ for in vivo CD4^+^ T‐cell activation, proliferation, and Th1‐cell differentiation during *Listeria monocytogenes* infection in mice

**DOI:** 10.1002/eji.201847961

**Published:** 2019-06-07

**Authors:** Sarah Frentzel, Konstantinos Katsoulis‐Dimitriou, Andreas Jeron, Ingo Schmitz, Dunja Bruder

**Affiliations:** ^1^ Institute of Medical Microbiology, Infection Prevention and Control, Infection Immunology Group, Health Campus Immunology, Infectiology and Inflammation Otto‐von‐Guericke University Magdeburg Magdeburg Germany; ^2^ Immune Regulation Group Helmholtz Centre for Infection Research Braunschweig Germany; ^3^ Systems‐oriented Immunology and Inflammation Research Group Helmholtz Centre for Infection Research Braunschweig Germany; ^4^ Institute for Molecular and Clinical Immunology, Health Campus Immunology, Infectiology and Inflammation Otto‐von‐Guericke University Magdeburg Magdeburg Germany

**Keywords:** CD4^+^ T cells, IκB_NS_, In vivo infection, *Listeria monocytogenes*, Th1 cell differentiation

## Abstract

Acquisition of effector functions in T cells is guided by transcription factors, including NF‐κB, that itself is tightly controlled by inhibitory proteins. The atypical NF‐κB inhibitor, IκB_NS,_ is involved in the development of Th1, Th17, and regulatory T (Treg) cells. However, it remained unclear to which extend IκB_NS_ contributed to the acquisition of effector function in T cells specifically responding to a pathogen during in vivo infection. Tracking of adoptively transferred T cells in *Listeria monocytogenes* infected mice antigen‐specific activation of CD4^+^ T cells following in vivo pathogen encounter to strongly rely on IκB_NS_. While IκB_NS_ was largely dispensable for the acquisition of cytotoxic effector function in CD8^+^ T cells, IκB_NS_‐deficient Th1 effector cells exhibited significantly reduced proliferation, marked changes in the pattern of activation marker expression, and reduced production of the Th1‐cell cytokines IFN‐γ, IL‐2, and TNF‐α. Complementary in vitro analyses using cells from novel reporter and inducible knockout mice revealed that IκB_NS_ predominantly affects the early phase of Th1‐cell differentiation while its function in terminally differentiated cells appears to be negligible. Our data suggest IκB_NS_ as a potential target to modulate specifically CD4^+^ T‐cell responses.

## Introduction

The development and function of immune cells are regulated by a variety of transcription factors including NF‐κB. NF‐κB acts as a molecular switch that regulates many immunological processes including cell proliferation, activation of immune cells, and regulation of inflammation 1. The activation of NF‐κB is controlled by inhibitory proteins, such as the classical NF‐κB inhibitor IκBα, which retains NF‐κB in the cytoplasm by masking the nuclear‐localization sequence 1. Next to the cytoplasmic inhibitory proteins, a second class of atypical NF‐κB inhibitors comprising Bcl3, IκBζ, IκBη, and IκB_NS_ exists in the nucleus, which either have activating or suppressing functions on NF‐κB‐mediated gene expression 2. The NF‐κB family of transcription factors contributes to Th1 differentiation. For instance, mice expressing a nondegradable IκBα mutant exhibit reduced IFN‐ɣ production and Th1 responses 3, 4. With respect to atypical NF‐κB inhibitors, Bcl‐3‐deficient mice exhibit impaired Th1 responses toward intracellular pathogens 5, 6. In contrast, T‐cell‐specific deletion of IκBζ results in increased IFN‐ɣ expression 7. IκBζ counteracts RelA/p65 activity at the *Ifng* locus and TGF‐β‐induced IκBζ represses *Ifng* promoter activity by reducing acetylation of histones associated with the *Ifng* locus 7. Similar to IκBζ, which interacts with chromatin‐modifying enzymes 8, 9, Bcl‐3 acts as a bridge to nuclear coregulators 10, 11.

IκB_NS_ was first described in thymocytes in the context of negative selection 12. IκB_NS_ is also expressed in different T‐cell subsets such as Th1 cells, regulatory T‐cell precursors, and Th17 cells 13, 14, 15. Of note, IκB_NS_^−/−^ mice exhibit reduced numbers of Tregs, because IκB_NS_ acts in concert with c‐Rel and p50 to regulate Foxp3 expression, thereby, mediating the transition of Treg precursors into mature Treg cells 14. In terms of T‐cell development and function, both IκB_NS‐_deficient CD4^+^ and CD8^+^ T cells, exhibit a proliferation defect upon in vitro TCR stimulation 13, 16. Moreover, IκB_NS_‐deficient T cells show decreased secretion of IL‐2 following in vitro stimulation and IκB_NS_^−/−^ Th1 cells produce less IFN‐γ 13, 15. Furthermore, IκB_NS_ is critical for the development of effector functions in Th17 cells both in vitro and in vivo 15. While together these data indicate a crucial role of IκB_NS_ in the development and function of different T‐cell subsets, no data are available on how IκB_NS_‐deficiency affects the activation, proliferation, and effector function of T cells specifically responding to a pathogen‐derived antigen during in vivo infection.

## Results and discussion

### IκB_NS_ fosters CD4^+^ T‐cell activation and Th1 cytokine induction during *Listeria monocytogenes* infection

**Figure 1:**
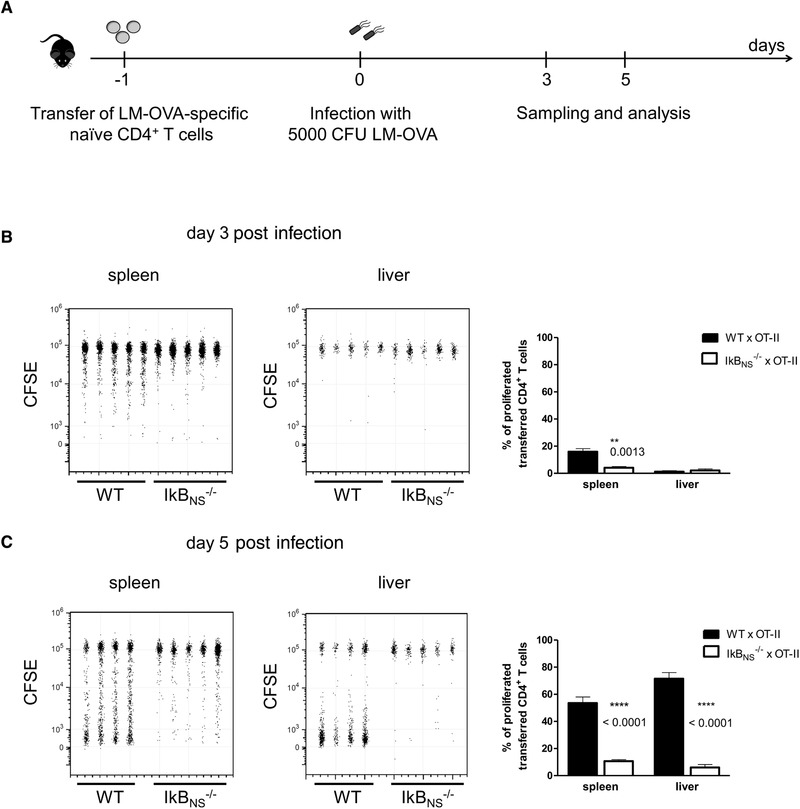
Proliferation of adoptively transferred LM‐OVA‐specific CD4^+^ T cells at different times post infection. (A) Experimental setup. 3 × 10^6^ CD4^+^ T cells from Thy1.1^+^ OT‐II x WT and OT‐II x IκB_NS_^−/−^ were transferred into C57BL/6 mice. One day post transfer recipients were infected with 5 × 10^3^ LM‐OVA. CD4^+^ T cells from spleen and liver were analyzed for CFSE loss at the indicated time post infection by flow cytometry. (B, C) Flow cytometry data are representative for two (day 3) or three (day 5) independent experiments with similar outcome with *n* = 4–5 individually analyzed mice/group and data were constrained to alive singlet Thy1.1^+^ CD4^+^ T cells and are shown in columns side‐by‐side in a concatenated qualitative dot plot in which each column represents data of an individual mouse. The summary plots are depicted as mean ± SEM of 4–5 individually analyzed mice/group and indicate the percentages of proliferated (CFSE^low^) transferred CD4^+^ T cells in spleen and liver samples. Statistics were performed using two‐tailed unpaired student's *t*‐test. **p < 0.01, ****p < 0.0001.

**Figure 2:**
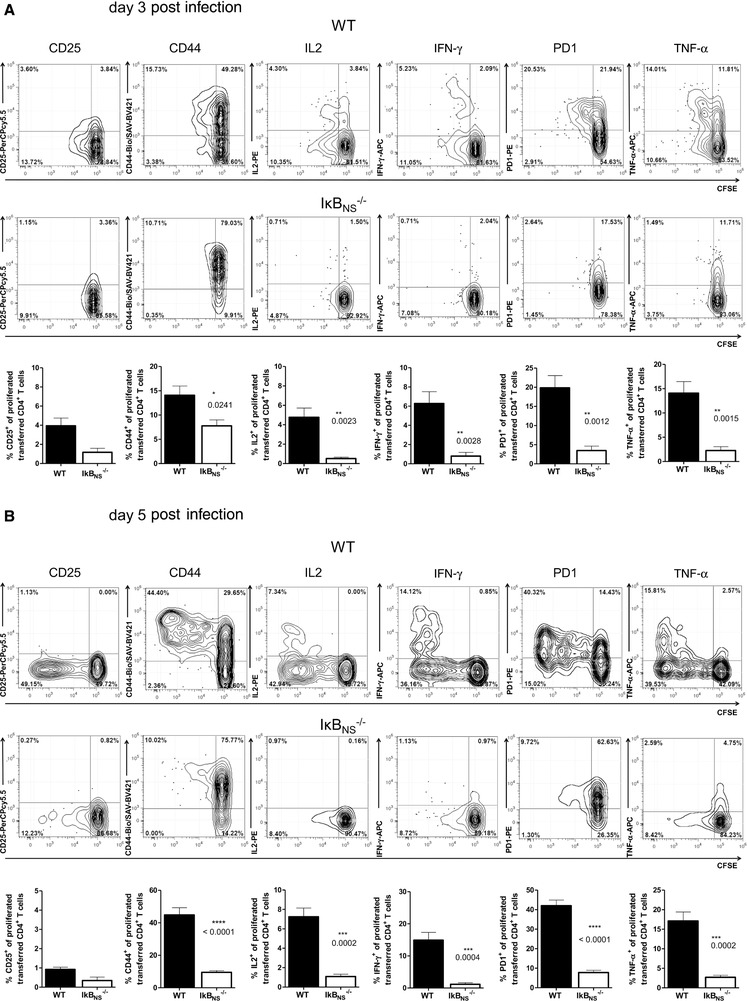
Phenotype of adoptively transferred LM‐OVA‐specific CD4^+^ T cells after LM‐OVA infection. Adoptive transfer of WT and IκB_NS_^−/−^ OT‐II CD4^+^ T cells was performed as described in Figure 1. (A) 3 and (B) 5 days post LM‐OVA infection splenic lymphocytes were analyzed by flow cytometry. Flow cytometry analysis was constrained to alive singlet Thy1.1^+^CD4^+^ T cells. Data are depicted as mean +/− SEM (*n* = 4–5 individually analyzed mice/group) from two (day 3) or three (day 5) independent experiments with similar outcome. Upper rows: representative contour plots with 5% probability with outliers for CD25, CD44, IFN‐γ, IL‐2, PD‐1, and TNF‐α versus CFSE from OT‐II CD4^+^ T cells. Lower rows: summary plots indicate percentages of marker‐positive T cells within the CFSE^low^ fraction. Statistics were performed using two‐tailed unpaired student‘s *t*‐test. *p < 0.05, **p < 0.01, ***p < 0.001.

To specify the role of IκB_NS_ in CD4^+^ T‐cell activation following in vivo pathogen recognition, we combined systemic infection with ovalbumin‐expressing *Listeria monocytogenes* (LM‐OVA) and adoptive transfer of IκB_NS_ sufficient or deficient TCR‐transgenic OT‐II CD4^+^ T cells (Fig. 1A). Analysis of reisolated cells revealed the first genotype‐specific differences in the spleen on day 3 post infection (Fig. 1B). Of note, by day 5, LM‐OVA‐specific IκB_NS_^+/+^ CD4^+^ T cells underwent extensive proliferation, which was impaired in CD4^+^ T cells lacking IκB_NS_ (Fig. 1B). Analyzing the proliferated CD4^+^ T cells for the expression of activation markers and Th1‐related effector cytokines revealed striking differences between the genotypes (Fig. 2). Here, lack of IκB_NS_ resulted in reduced frequency of LM‐OVA‐specific CD4^+^ T cells expressing the activation markers CD44 and PD‐1, as well as the Th1‐effector cytokines IFN‐γ, IL‐2, and TNF‐α. We conclude that IκB_NS_ is required for CD4^+^ T‐cell activation and expansion in in vivo infectious settings and is critically involved in Th1‐cell differentiation.

### IκB_NS_ affects the early phase of Th1‐cell differentiation

**Figure 3:**
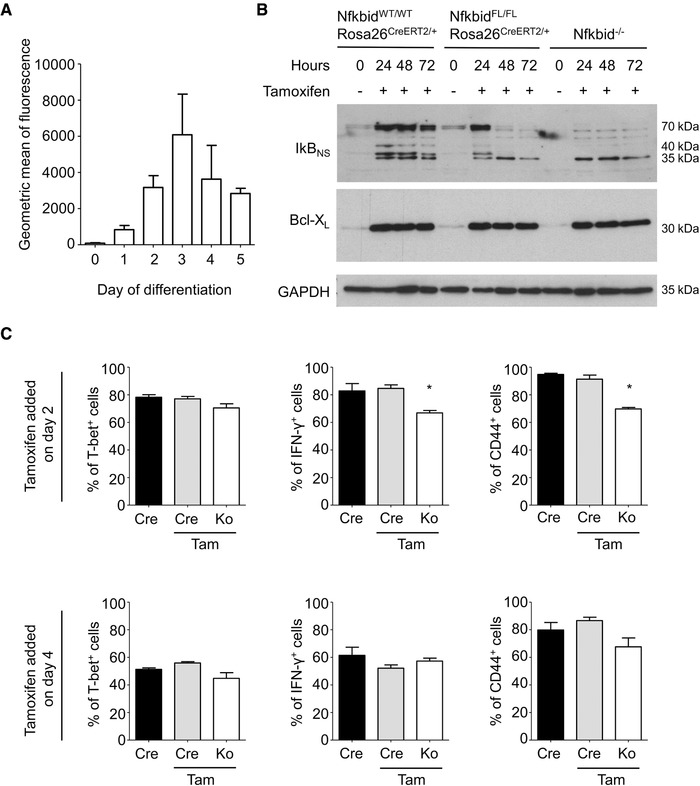
Conditional deletion of Nfkbid during Th1 differentiation. (A) Nfkbid promoter activity in CD4^+^ T cells from Nfkbid^LacZ^ mice during Th1 differentiation day 0 (unstimulated) and day 1–5 following α‐CD3/α‐CD28 stimulation in the presence of α‐IL‐4 and α‐IL‐12. Data are shown as mean +/− SEM (*n* = 3) and are representative of three independent experiments with *n* = 1 mouse per experiment. Chromophores: pacific blue for CD4 and FITC for lacZ. (B) Kinetic of IκB_NS_ protein expression in CD4^+^ T cells from spleen and lymph nodes after tamoxifen administration. Nfkbid^WT/WT^Rosa26^CreERT2/+^: WT control; Nfkbid^Fl/FL^Rosa26^CreERT2/+^: conditional deletion of Nfkbid; IκB_NS_^−/−^: negative control. Cells were stimulated with anti‐CD3 (2 µg/mL plate bound), anti‐CD28 (2 µg/mL in suspension), and IL‐2 (50 ng/mL). Data are representative of two independent experiments with *n* = 2 mice per experiment. (C) Conditional deletions of Nfkbid during Th1 differentiation. Cre: Nfkbid^WT/WT^Rosa26^CreERT2/+^ sample without addition of tamoxifen; Cre Tam: same sample with addition of tamoxifen. Ko: tamoxifen‐treated Nfkbid^Fl/FL^Rosa26^CreERT2/+^ sample (conditional deletion). Data are shown as mean +/− SEM and are pooled from six (IFN‐γ) or three (T‐bet, CD44) independent experiments with *n* = 1 mouse per condition (Cre, Ko) per experiment. Statistics were performed using two‐tailed unpaired student‘s *t*‐test (Mann–Whitney test). The *p* values are 0.026 for IFN‐γ and 0.045 for CD44. Chromophores: APC for IFN‐γ, FITC for T‐bet, and PE‐Cy7 for CD44. Gating: see Supporting Information Figure 7.

To investigate in more detail IκB_NS_ dependency of Th1‐cell differentiation, we used the newly generated reporter mouse Nfkbid^lacZ^ that contains a LacZ cassette within the IκB_NS_‐encoding *Nfkbid* gene and expresses ß‐galactosidase under the control of the *Nfkbid* promoter. After confirmation that the Nfkbid^lacZ^ mouse represents a faithful reporter to quantify *Nfkbid* gene expression (Supporting Information Fig. 1), we analyzed the kinetics of *Nfkbid* promoter activity under Th1‐polarizing conditions. Promoter activity increased until day 3 following T‐cell activation (Fig. 3A), suggesting that IκB_NS_ is especially important for the early phase of Th1‐cell differentiation. To further confirm this, we utilized Nfkbid^FL/FL^ × Rosa^CreERT2^ mice, which allow for targeted deletion of *Nfkbid* at time of interest following T‐cell stimulation. After confirming complete loss of IκB_NS_ in CD4^+^ T cells within 48 h following stimulation (Fig. 3B), we evaluated at which phase of Th1‐cell differentiation IκB_NS_ is required. Both, IFN‐γ and CD44 expression were reduced in IκB_NS_^−/−^ CD4^+^ T cells when IκB_NS_‐deficiency was induced early (day 2) during Th1‐cell differentiation (Fig. 3C) but were not affected by adding tamoxifen on day 4 (Fig. 3C). Notably, the expression of T‐bet, which is crucial for Th1‐cell differentiation 17 was not affected in the absence of IκB_NS_ (Fig. 3C), suggesting that other early factors of Th1‐cell differentiation are affected. Since IFN‐ɣ amplifies Th1‐cell differentiation, we suspect that the reduced IFN‐ɣ expression in the absence of IκB_NS_ is responsible for reduced Th1 responses. As IκBζ and IκB_NS_ have opposing activities on IL‐6 expression in macrophages 16, 18, it is tempting to speculate that a similar counteracting function operates in Th1 cells with respect to IFN‐ɣ expression.

Another hint regarding a potential mechanism underlying the impact of IκB_NS_ on Th1‐cell differentiation comes from data published by Touma et al., who showed that the F‐box protein family member Fbxo17 is highly overexpressed in resting and activated IκB_NS_^−/−^ T cells 13, a finding we confirmed in OT‐II CD4^+^ T cells (data not shown). F‐box proteins are the substrate‐binding subunits of SCF E3 ubiquitin ligases involved in regulation of cell cycle and proliferation 19. Fbxo17 regulates proteasomal degradation of glycogen synthase kinase‐3β (GSK3β) 20. Since GSK3 isoforms are crucial for Th1 differentiation 21, increased Fbxo17 expression in IκB_NS_^−/−^ T cells might explain impaired Th1 differentiation. The exact mechanism of IκB_NS_ and/or Fbxo17 in Th1 differentiation remains, however, unresolved and will be the focus of future studies.

*Listeria monocytogenes* induces a Th1‐dominated response with almost no Th17 cells and Th2‐cell responses even actively being suppressed by pathogen‐derived factors 22, 23. In line with data obtained in the in vitro Th1 differentiation, IκB_NS_‐deficient CD4^+^ T cells failed to upregulate CD44 and to produce the Th1‐effector cytokines IFN‐γ, IL‐2, and TNF‐α following in vivo pathogen encounter (Fig. 2). IκB_NS_‐dependency of IL‐2 and IFN‐γ production in CD4^+^ T cells has been described before 13 and we recently showed that IκB_NS_‐deficiency impairs CD4^+^ T‐cell proliferation during in vitro Th‐cell differentiation and is critically involved in the development of Th1 and Th17 cells 15. However, the impact of IκB_NS_ on T‐cell differentiation appears to be context dependent. While EAE induction in IκB_NS_^−/−^ mice did not affect the Th1‐cell pool 24, the DSS colitis model uncovered increased IFN‐γ expression in IκB_NS_^−/−^ CD4^+^ T cells 15, 16. In contrast, intestinal infection of IκB_NS_‐deficient mice with *Citrobacter rodentium* resulted in reduced frequencies of IFN‐γ‐producing CD4^+^ T cells in spleen but not in colon and local lymph nodes 15. Of note, the present study is fundamentally different since we specifically track T cells responding to pathogen‐derived antigen in mice exhibiting an IκB_NS_‐sufficient immune system.

In line with published data obtained with polyclonal T cells 13, we also found OT‐I CD8^+^ IκB_NS_^−/−^ T‐cell proliferation to be impaired following in vitro antibody‐induced TCR stimulation (data not shown) while CD8^+^ T‐cell proliferation induced by antigen‐specific activation following in vivo pathogen encounter does not depend on IκB_NS_ (Supporting Information Fig. 2). Moreover, with the exception of TNF‐α, IκB_NS_‐deficiency had no or only transient effect on the expression of all other markers analyzed (Supporting Information Fig. 3). Data from adoptive transfers suggest that IκB_NS_ might affect cytotoxic function of CD8^+^ T cells during the early phase of infection, which is likely to be compensated by the WT adaptive immunity in the later infection phase (Supporting Information Fig. 4A). This was supported by LM‐OVA infections in WT and conventional IκB_NS_^−/−^ mice that neither revealed differences in bacterial elimination (Supporting Information Fig. 4B) nor uncovered defects in the establishment of cytotoxic T‐cell responses from the polyclonal TCR pool (Supporting Information Fig. 4C). This at least in part excludes that the observed effects in IκB_NS_^−/−^ CD8^+^ T cells are due to the transgenic OT‐I TCR specific for the model antigen expressed by LM‐OVA. We hypothesize that the effects of IκB_NS_‐deficiency in CD8^+^ T cells that are observed in vitro are compensated in vivo by the presence of host‐derived proinflammatory cytokines that are produced during infection. This is in line with our observation that genotype‐dependent differences in the activation pattern of CD8^+^ T cells are only transient. Interestingly, the percentage of TNFα‐producing LM‐OVA‐specific IκB_NS_^−/−^ CD8^+^ T cells was significantly higher compared to IκB_NS_^+/+^ CD8^+^ T cells. There is evidence that IκB_NS_ may function as suppressor of TNF‐α 25. To rule out the possibility that the fraction of TNF‐α^+^ CD8^+^ T cell is per se higher in IκB_NS_^−/−^ mice, we analyzed its expression at 1 day post infection revealing even more TNF‐α^+^ CD8^+^ T cells in the IκB_NS_^+/+^ group (data not shown). Nevertheless, despite distinct changes in the activation pattern and kinetics, IκB_NS_^−/−^ CD8^+^ T cells are fully capable of producing IFN‐γ and mice with IκB_NS_‐deficient T cells are competent in developing cytotoxic T‐cell responses against LM‐OVA (Supporting Information Fig. 4).

## Concluding remarks

While being largely dispensable for the in vivo acquisition of effector function in CD8^+^ T cells, we unveiled IκB_NS_ to play a pivotal role in the activation, expansion, and Th1‐cell differentiation of CD4^+^ T cells following in vivo pathogen encounter. In terms of timing, IκB_NS_ affects the early phase of Th1‐cell differentiation but is dispensable in terminally differentiated Th1 cells.

## Material and methods

### Animals

IκB_NS_^−/−^ mice (B6.129/SV‐NFKBID(tm1Clay)) 14, OT‐I x CD90.1 26, OT‐II x CD90.1 27, and IκB_NS_^‐/‐^ mice crossed on OT‐I and OT‐II were bred and maintained under specific pathogen‐free conditions at the animal facilities of the Helmholtz Centre for Infection Research Braunschweig, Germany and the University Hospital Magdeburg, respectively. For genotyping of mice, see Supporting Information Figure 5. Nfkbid^lacZ^ (Nfkbid^tm1a(EUCOMM)Wtsi^) reporter mice were obtained from EUCOMM. Nfkbid^Fl^ mice were generated by crossing Nfkbid^lacZ^ with FLP recombinase expressing mice and offsprings were crossed to B6.129‐*Gt(ROSA)*26Sor*^tm1(cre/ERT2)Tyj^* ^/^ *^J^* mice 28 to create an inducible IκB_NS_^−/−^ mouse. All experiments were approved by the Landesamt für Verbraucherschutz, Sachsen‐Anhalt (AZ 42502‐2‐1242).

### Adoptive T‐cell transfers

OVA‐specific CD4^+^ and CD8^+^ T cells were isolated using CD4^+^ and CD8^+^ T‐cell isolation kits and an autoMACS (Miltenyi Biotec). Purity of cells was ≥90%. Cells were stained with CFSE (2.5 µM, Thermo Fisher Scientific) and 3 × 10^6^ cells were injected in 200 µL sterile PBS (Gibco) into the tail vein of C57BL/6J recipient mice (Janvier Labs, Le Genest‐Saint‐Isle, France or Envigo, NM Horst, The Netherlands).

### Bacterial infection

OVA‐expressing *L. monocytogenes* (LM‐OVA, strain 10403S) were grown overnight (37°C, 180 rpm) in BHI broth (BD Biosciences). A 1:5 dilution was prepared with fresh BHI and after 3 h bacteria were harvested and diluted in sterile PBS to establish an infection dose of 5 × 10^3^ CFU/mouse. To determine CFU in spleen and livers organs were homogenized in 0.2% IGEPAL CA‐630 (Sigma‐Aldrich) lysis buffer and serial dilutions were plated on BHI agar plates to quantify colonies after incubation at 37°C for 24 h.

### Cell preparation

Spleens and livers were squeezed through 100 µm cell strainers (Falcon), washed with PBS (300 × *g*, 10 min, 4°C) followed by erythrocyte lysis. Splenocytes were passed through a 30 µm cell strainer and resuspended in PBS. Liver cells were separated by density centrifugation in a 35% mixture of Easycoll (Biochrom) and PBS. After centrifugation (360 × *g*, 20 min, RT) cells were washed and resuspended in PBS.

### Flow cytometric analyses

Flow cytometric analyses were performed in adherence to the “Guidelines for the use of flow cytometry and cell sorting in immunological studies” 29. Cells were stained with anti‐CD16/32 (Fc‐block) (BioLegend) and with Fixable Viability Dye‐eFluor 780 (eBioscience) to exclude dead cells. After washing, cells were incubated with the antibody mixture containing CD8a‐BV510 (53–6.7) or CD4‐BV510 (GK1.5), CD90.1‐Pe‐Cy7 (OX‐7), CD44‐Biotin (IM7), CD25‐PerCP‐Cy5.5 (PC61), and PD1‐PE (29F.1A12) (all BioLegend). In case of biotinylated antibodies, a second step with streptavidin‐BV421 (BioLegend) was performed. Transgenic T cells were identified by FACS as shown in Supporting Information Figure 6. For detection of intracellular cytokines, cells were stimulated with 1 µg/mL OVA_323‐339_ or OVA_257‐264_ peptide (synthesized at Helmholtz Centre for Infection Research Braunschweig, Germany). After 1 h brefeldin A (5 µg/mL, BioLegend) was added. After another 4 h, cells were washed and stained for extracellular markers and fixed with 2% PFA. Subsequently, cells were permeabilized (0.1% IGEPAL CA‐630 (Sigma‐Aldrich), 4 min, 4°C), washed and stained with anti‐IFN‐γ‐APC (XMG1.2), anti‐IL‐2‐PE (JES6‐5H4) or anti‐TNF‐α‐APC (MPC‐XT22) (BioLegend) for 30 min at 4°C. IκB_NS_‐dependency of cytotoxic function in CD8^+^ T cells was determined as described before 30. Cells were analyzed with a BD FACS Canto II, BD LSR II (BD Biosciences) or an Attune NxT Acoustic Focusing Cytometer (Thermo Fisher Scientific).

### Th1‐cell differentiation

Naïve CD4^+^CD62L^+^CD25^−^ T cells were isolated from spleens and lymph nodes by cell sorting (BD FACS Aria II (BD Biosciences) or Moflo (Beckman and Coulter)). Th1 differentiation was performed as described before 15. In case conditional deletion of Nfkbid was performed, 1 µM (Z)‐4‐Hydroxytamoxifen (Sigma–Aldrich) was added on day 2 and 4. On day 4 and 6, cells were restimulated with PMA (10 ng/mL) and ionomycin (1 µg/mL, both Sigma–Aldrich) for 4 h. After 2h stimulation, brefeldin A (10 µg/mL, Sigma–Aldrich) was added and subsequently cells were stained with LIVE/DEAD fixable blue (Life Technologies), for CD4‐Pacific Blue (RMA4‐5, BioLegend) and CD44‐PE‐Cy7 (IM7, eBioscience). Afterwards, the cells were fixed with Foxp3 staining buffer (Miltenyi Biotec) and stained for IFN‐γ‐APC (XMG1.2, BioLegend) and T‐bet‐FITC (4B10, BioLegend) according to manufacturer's recommendations.

### Western blot analysis

Cells were lysed and immunoblot was performed as previously described 15. The following primary antibodies were used: IκB_NS_ (rabbit, β‐actin (AC‐74, Sigma–Aldrich) 31, GAPDH (1E6D9, Proteintech), Bcl‐X_L_ (clone 4, BD Biosciences).

### Statistics

Results are expressed as mean ± SEM. Statistical analyses were performed with the GraphPad Prism 5.4 Software (La Jolla, CA, USA) and a *p*‐value below 0.05 was considered significant.

## Conflict of interest

The authors declare no commercial or financial conflict of interest.

**Abbreviation**
GSK3β: glycogen synthase kinase‐3β

## Supporting information

Supporting Information
