# Supplementary material for: Essential role of IκBNS for in vivo CD4+ T‐cell activation, proliferation, and Th1‐cell differentiation during Listeria monocytogenes infection in mice
Source: Eur J Immunol. 2019 Jun 7;49(9):1391–8. doi: 10.1002/eji.201847961 (PMC6771600; doi:10.1002/eji.201847961)
Supplement: Supplementary file 1 — Supporting Information [file EJI-49-1391-s001.docx]

**Supporting Information**

**Figure Legends**

**Supporting Figure 1:** ***Nfkbid* promoter activity is induced shortly after CD4^+^ T cell stimulation.** (A) Lymphocytes from peripheral lymph nodes of Nfkbid^LacZ/+^ reporter and Nfkbid^+/+^ wild type mice were isolated and 5 x 10^6^ cells were stimulated with PMA (10 µg/mL) and ionomycin (1 µg/mL). At indicated times post stimulation, 5 x 10^5^ cells were stained with fluorescein Di-ß-D-galactopyranosid (FDG) as substrate for the reporter enzyme ß-galactosidase encoded by the lacZ cassette. For the detection of ß-galactosidase activity the FluoReporter® lacZ flow cytometry kit (Molecular Probes) was used according to manufacturer’s instructions. Nfkbid promoter activity was analyzed by flow cytometry through determination of the intensity of fluorescein, which is produced after cleavage of FDG (upper panel). The remaining cells were lysed and the protein expression of IκB_NS_ was determined by western blot with β-actin as loading control (lower panel). Data are shown as mean ±SEM (n=2) and are representative of 2 independent experiments with n=1 mouse per experiment. The western blot is representative of two independent experiments. (B) Flow cytometric analyses of the basal Nfkbid promoter activity in CD4^+^ T cells. The FACS plots shown are representative of 2 independent experiments with n=1 mouse per experiment. (C) Flow cytometric analysis of the Nfkbid promoter activity in CD4^+^ T cells upon stimulation with plate-bound anti-CD3 (10 µg/mL) for 3 h. The FACS plots shown are representative of 2 independent experiments with n=1 mouse per experiment.

**Supporting Figure 2: Proliferation of adoptively transferred LM-OVA-specific WT x OT-I and IκB_NS_^-/-^ x OT-I CD8^+^ T cells at different times post systemic LM-OVA infection.** (A) Schematic overview about the experimental set-up. CD8^+^ T cells from WT x OT-I and IκB_NS_^-/-^ x OT-I with Thy1.1 congenic background were MACS purified, CFSE-labeled and 3 x 10^6^ T cells were adoptively transferred into naive C57BL/6 recipient mice. One day post transfer, recipient mice were infected with LM-OVA and sacrificed at the indicated time post infection. Lymphocytes from spleen and liver were isolated and the CFSE loss of the CD8^+^ T cells was analyzed by FACS. (B) FACS data from one independent experiment with n=5-6 individually analyzed mice/group were constrained to alive singlet Thy1.1^+^ CD8^+^ T cells and are shown in columns side-by-side in a concatenated qualitative dot plot in which each column represents data of an individual mouse.

**Supporting Figure 3: Phenotype of adoptively transferred LM-OVA-specific WT x OT-I and IκB_NS_^-/-^ x OT-I CD8^+^ T cells after LM-OVA infection.** WT (black bars) and IκB_NS_^-/-^ (white bars) OT-I CD8^+^ T cells with Thy1.1 congenic background were MACS-purified, CFSE-labeled and 3 x 10^6^ T cells were adoptively transferred into naive C57BL/6 recipient mice, respectively. One day post transfer recipient mice were i.v. infected with 5 x 10^3^ CFU LM-OVA. (A) 3 and (B) 5 days post infection recipient mice were sacrificed and splenic lymphocytes were stained with antibodies against CD25, CD44, IL2, IFNγ, PD1 and TNFα and re-analyzed by FACS. Prior to intracellular staining of IFNγ, IL2, TNFα, the cells were re-stimulated with 1.0 µg/mL OVA_257-264_ for 5 h and brefeldin A was added to the culture for the last 4 h of incubation. FACS data were constrained to alive singlet Thy1.1^+^ CD8^+^ T cells. Data are depicted as mean ± SEM (n=5 individually analyzed mice/group) of one independent experiment. Upper rows: Representative contour plots with 5% probability with outliers for CD25, CD44, IL2, IFNγ, PD1 and TNFα vs. CFSE from OT-I CD8^+^ T cells. Lower row: Summary plots indicate percentages of CD25^+^, CD44^+^, IL2^+^, IFNγ^+^, PD1^+^ and TNFα^+^ T cells within the CFSE low fraction. Statistics were performed using two-tailed unpaired student‘s t-test. * p < 0.05, ** p < 0.01, *** p < 0.001.

**Supporting Figure 4: *In vivo* analysis of LM-OVA induced CD8^+^ T cell cytotoxicity.** (A)  Bacterial burden in spleens of LM-OVA infected C57BL/6 mice which received OT-I CD8^+^ T cells from WT (▲) or IκB_NS_^-/-^ (□) donor mice. Vertical line represents the mean of each group with n=4-6 individually analyzed mice/group of one independent experiment. Statistical analyses were performed with 2-way ANOVA with Bonferroni‘s post-test. (B) WT (▲) and conventional IκB_NS_^-/-^ (□) mice (n=4-5/group) were infected with 1 x 10^4^ CFU LM-OVA and bacterial burden was determined at indicated times post infection. Vertical line represents the mean of each group. Statistical analyses were performed with 2-way ANOVA with Bonferroni‘s post-test. (C) Splenocytes of C57BL/6 mice were pulsed with either 1 µg/mL or 0.1 µg/mL OVA_257-264_ peptide or were left unpulsed as control cell fraction. Peptide-pulsed cell fraction was stained with a high concentration of CFSE (2.5 µM) and the control fraction was stained with a low concentration of CFSE (0.25 µM). Both cell fractions were mixed in a 1:1 ratio and were injected intravenously into WT and IκB_NS_^-/-^ mice 9 days post LM-OVA infection. The capacity of cytotoxic T cells to lyse the transferred target cells was determined 12 h post injection by FACS though assessing the relative amounts of CFSE^high^ and CFSE^low^ cells. Vertical line represents the mean of each group with n=4-5 individually analyzed mice/group per point in time of one independent experiment.

**Supporting Figure 5: Representative gating strategy for genotyping of OT-I and OT-II TCR transgenic mice from blood samples.** Mice were bled retrobulbar with capillary blood collection tubes and blood samples were prepared with BD FACS™ Lysing solution (BD Biosciences, USA) according to the manufacturer’s recommendation. Cells were pre-gated on leukocytes by FSC-Area vs. SSC-Area and doublets were excluded by FSC-Area vs. FSC-Height gating. CD4^+^ (A) and CD8^+^ (B) T cells were detected by staining with CD4-APC (RM4-5) or CD8-APC (53-6.7) (both obtained from BioLegend). Transgenic T cells were further analyzed by staining the according TCR chains TCR Vα2-FITC (B20.1) and TCR β5.1/5.2-PE (MR9-4) and the congenic markers CD90.1-PE-Cy7 (OX-7) and CD90.2- PerCP (30-H12) (all obtained from BioLegend). (C) For IκB_NS_ genotyping, small biopsies of the ears were processed with the KAPA Mouse Genotyping Hot Start kit (Sigma-Aldrich) according to the manufacturer’s recommendation. For genotyping the following primers were used IκB_NS__Neo AAG CGC ATG CTC CAG ACT GCC TT, IκB_NS__rev CAT TTA GTG CCC CTG GAC AT, IκB_NS__fwd CTC CTC CCA GGC TGT GTT TA.

**Supporting Figure 6: Representative gating strategy for the analysis of transferred OVA-specific CD4^+^ and CD8^+^ T cells into LM-OVA infected C57BL/6 recipient mice.** Leukocytes were pre-gated by SSC-Area vs. FSC-Area properties. Doublets were excluded by FSC-Height vs. FSC-Area gating and dead cells were excluded by live/dead discrimination using Fixable Viability Dye-eFluor780 (eBioscience). Transferred cells were identified by staining for (A) CD4-BV421 (GK1.5) or (B) CD8-BV421 (53-6.7) together with staining for the congenic marker CD90.1 (Thy1.1)-PE-Cy7 (OX-7).

**Supporting Figure 7: Gating strategy for flow cytometric analysis of Th1 differentiation.** For all Th1 cell differentiation experiments the gating was performed on T cells, followed by doublet exclusion, then live cell selection and finally gating on CD4^+^ cells. After that, different markers were stained in live CD4^+^ T cells, according to the experiment. (A) For visualizing Nfkbid promoter activity (Full results shown in figure 3A) the mean of fluorescence intensity in the FITC channel was used. (B) This is the staining and gating for measuring the percentages of IFN-γ+, T-bet+ and CD44+ cells, (Full results shown in figure 3C).

**Supporting Figure 1**

**
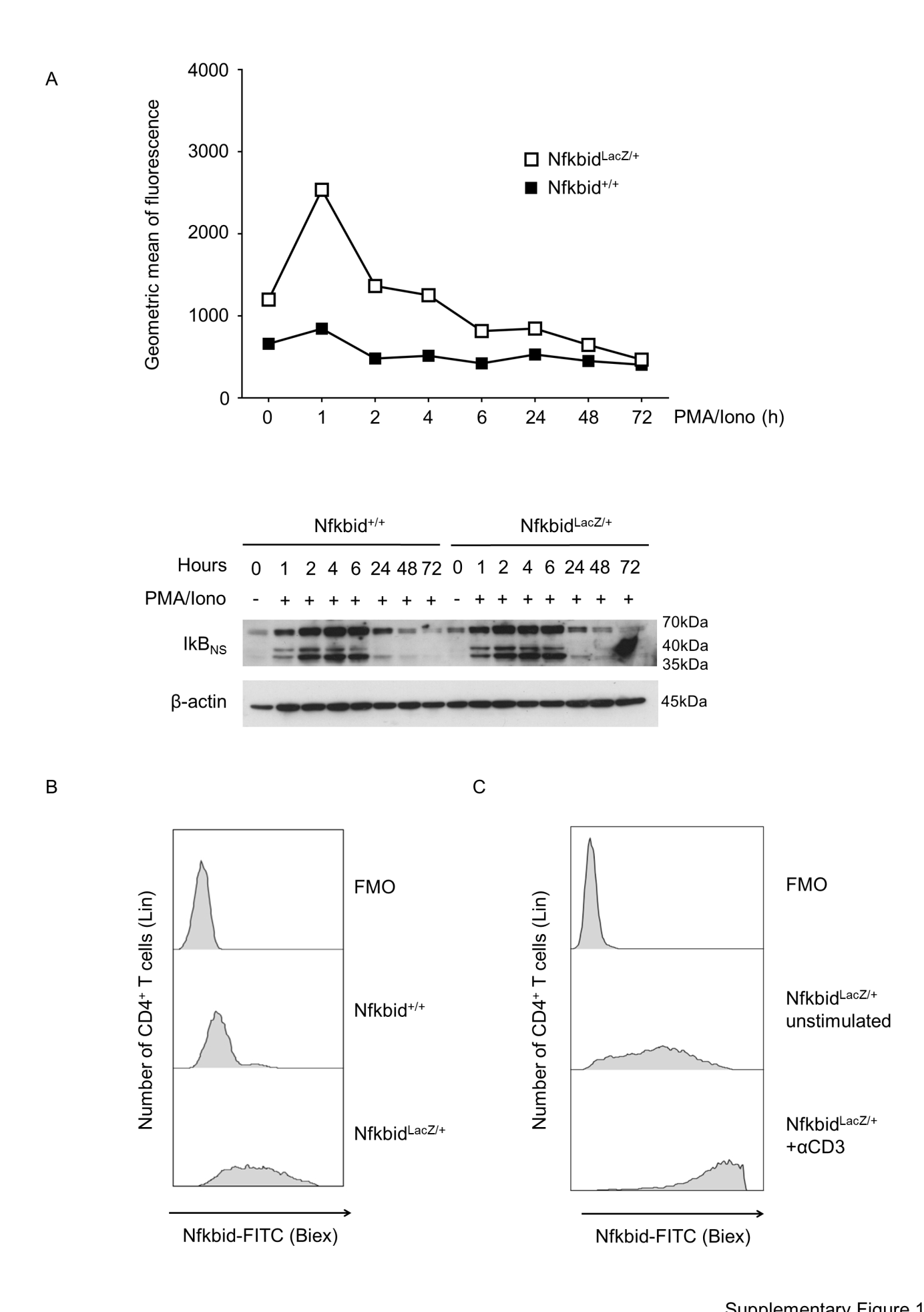
**

**Supporting Figure 2**

**
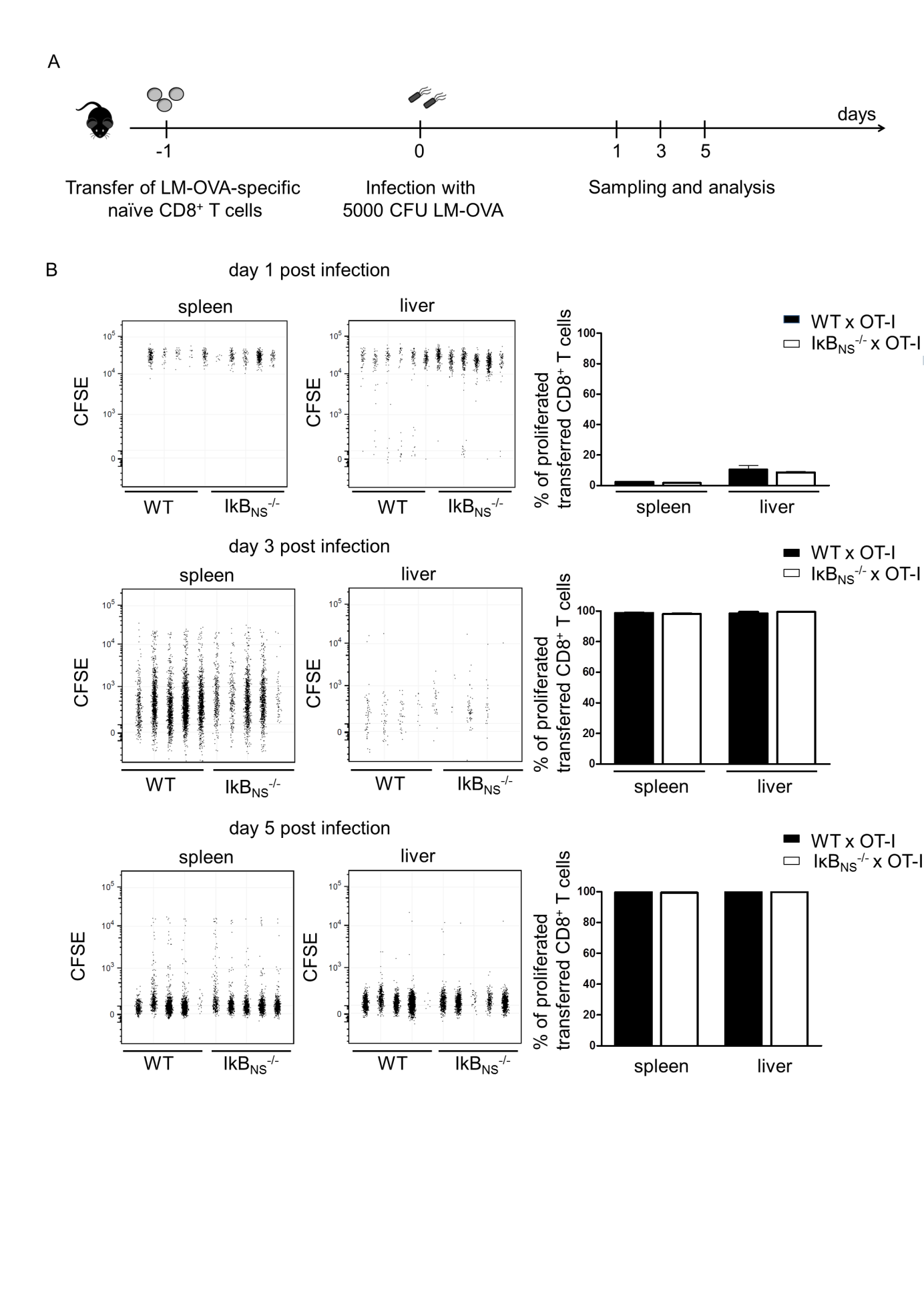
**

**Supporting Figure 3**

**
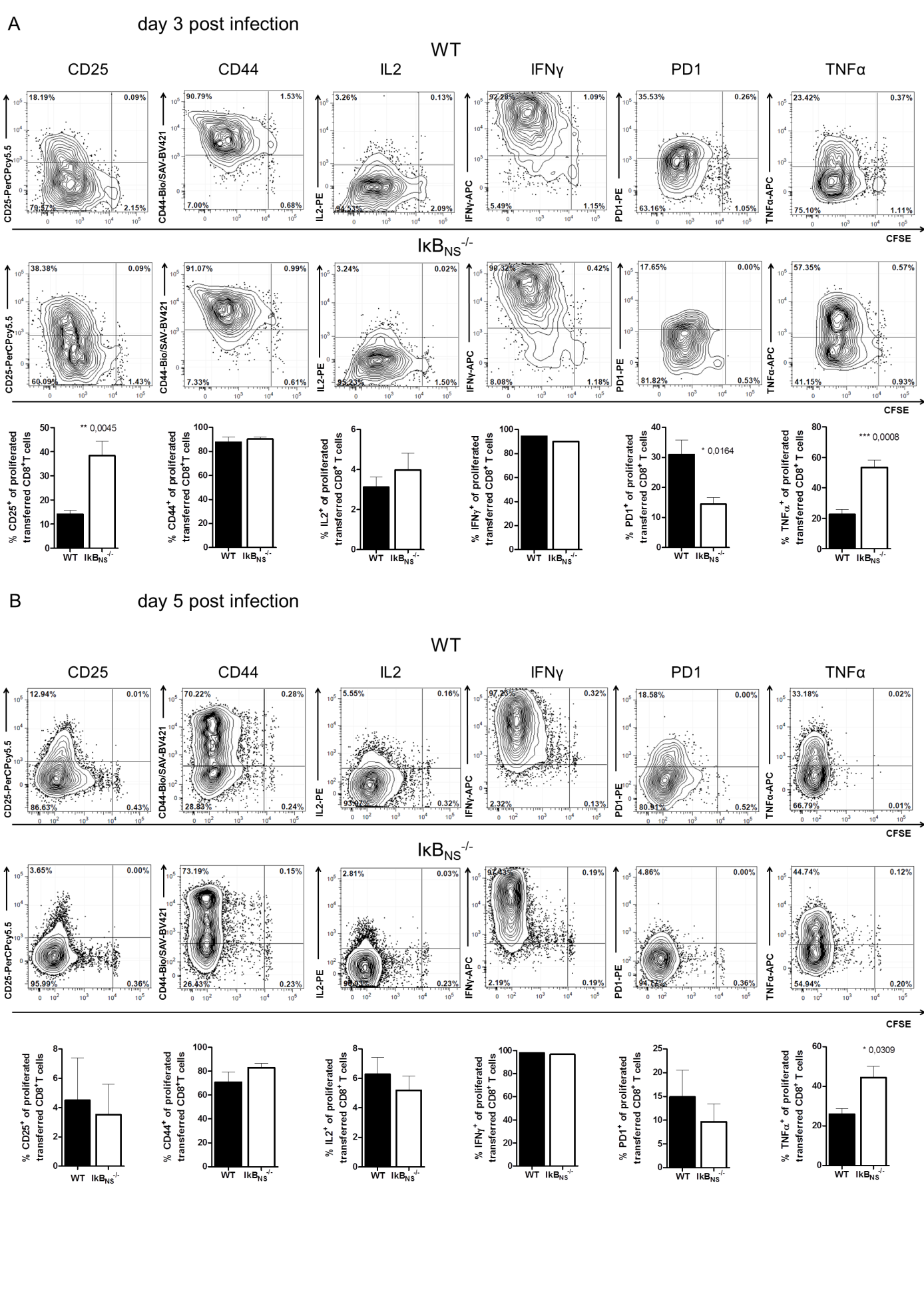
**

**Supporting Figure 4**

**
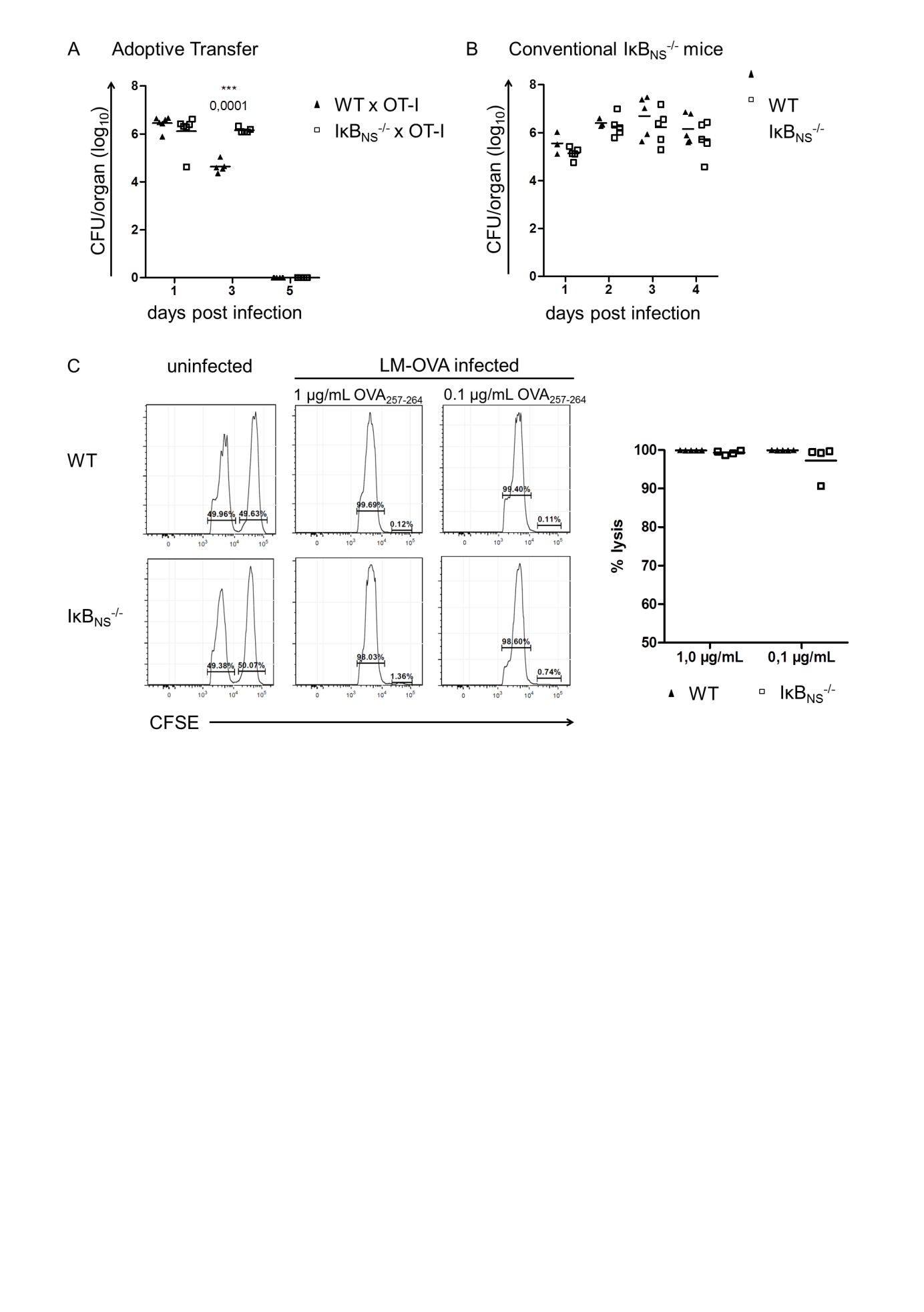
**

**Supporting Figure 5**

**
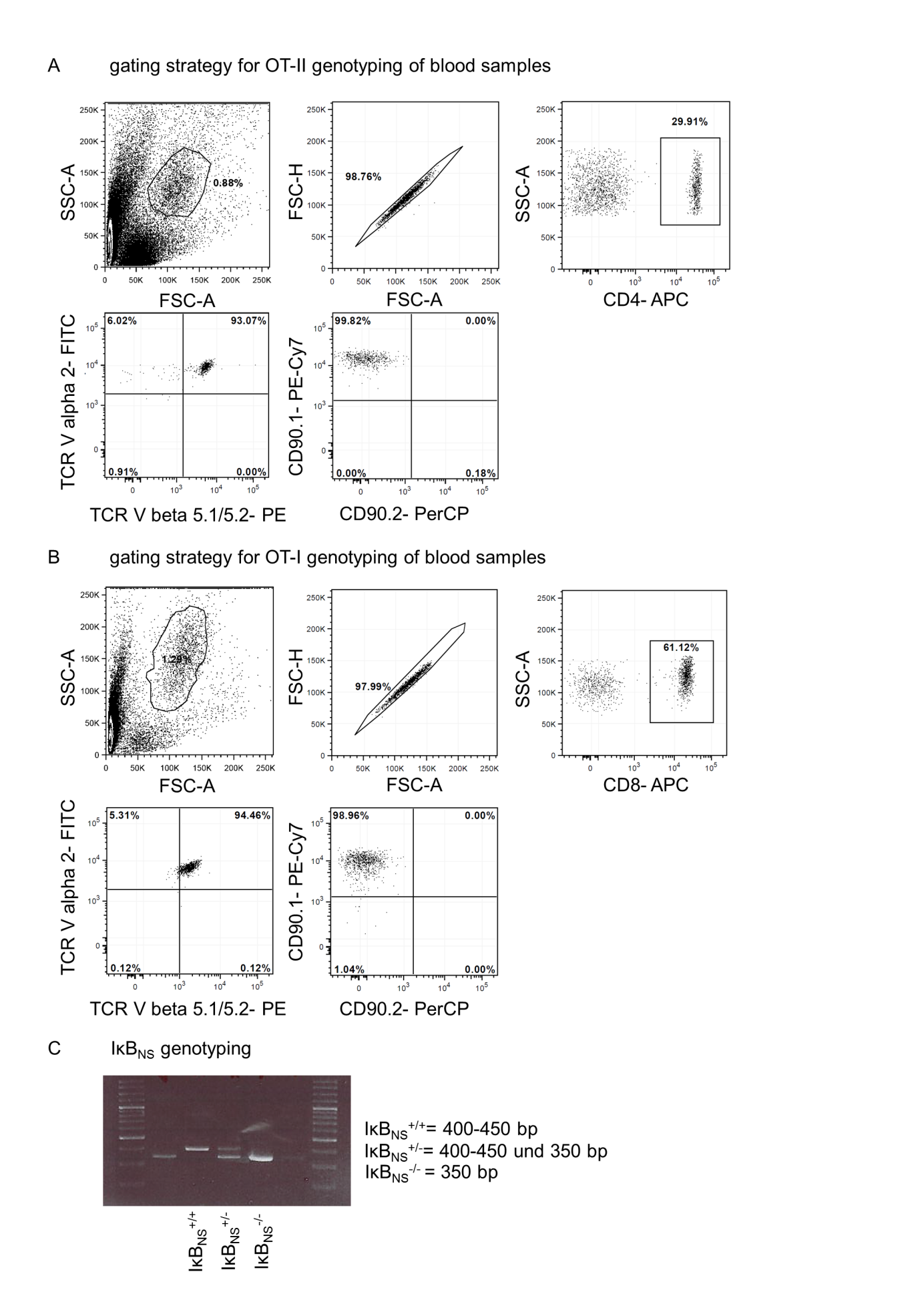
**

**Supporting Figure 6**

**
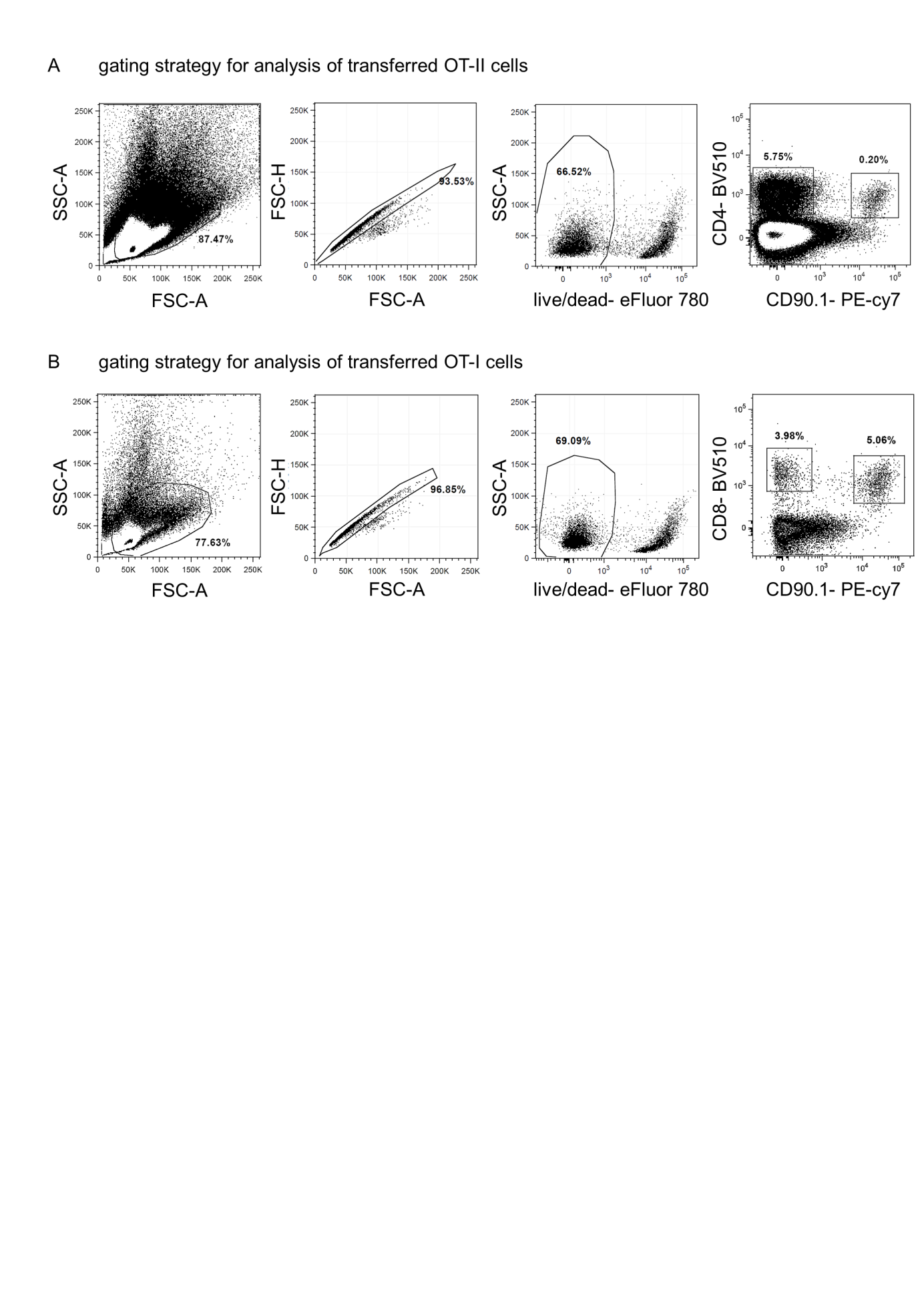
**

**Supporting Figure 7**

**
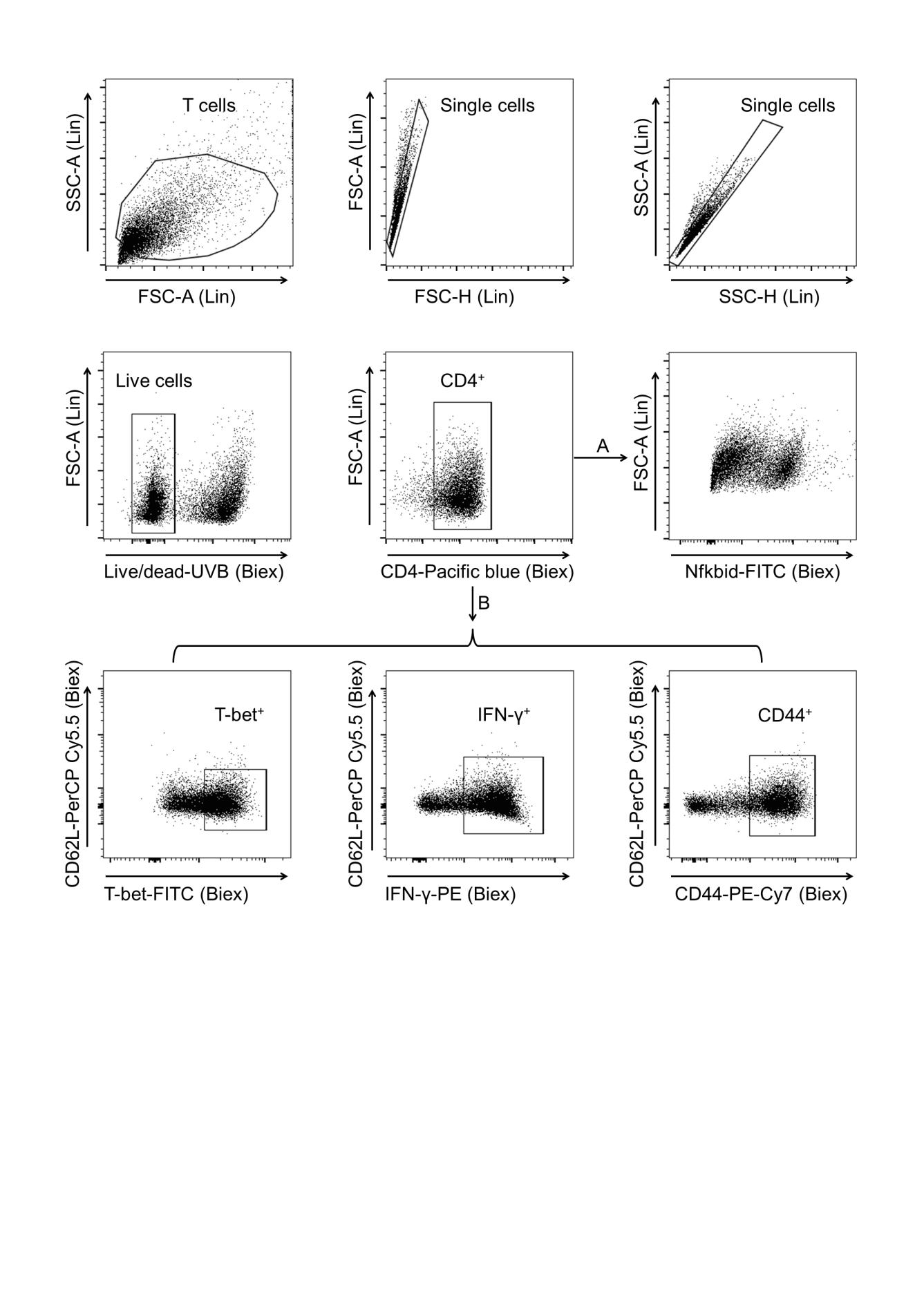
**
